# Critical role of bacterial dissemination in an infant rabbit model of bacillary dysentery

**DOI:** 10.1038/s41467-019-09808-4

**Published:** 2019-04-23

**Authors:** Lauren K. Yum, Mariana X. Byndloss, Sanford H. Feldman, Hervé Agaisse

**Affiliations:** 1 Department of Microbiology, Immunology, and Cancer Biology, University of Virginia, Charlottesville, VA USA; 2 Department of Pathology, Microbiology, and Immunology, Vanderbilt University Medical Center, Nashville, TN USA; 3 Center for Comparative Medicine, University of Virginia, Charlottesville, VA USA

**Keywords:** Bacterial pathogenesis, Cellular microbiology, Pathogens

## Abstract

The bacterial pathogen *Shigella flexneri* causes 270 million cases of bacillary dysentery (blood in stool) worldwide every year, resulting in more than 200,000 deaths. A major challenge in combating bacillary dysentery is the lack of a small-animal model that recapitulates the symptoms observed in infected individuals, including bloody diarrhea. Here, we show that similar to humans, infant rabbits infected with *S. flexneri* experience severe inflammation, massive ulceration of the colonic mucosa, and bloody diarrhea. T3SS-dependent invasion of epithelial cells is necessary and sufficient for mediating immune cell infiltration and vascular lesions. However, massive ulceration of the colonic mucosa, bloody diarrhea, and dramatic weight loss are strictly contingent on the ability of the bacteria to spread from cell to cell. The infant rabbit model features bacterial dissemination as a critical determinant of *S. flexneri* pathogenesis and provides a unique small-animal model for research and development of therapeutic interventions.

## Abstract (web summary)

The bacterial pathogen *Shigella flexneri* causes bacillary dysentery (bloody diarrhoea). Here, Yum et al. present an infant rabbit model of *S. flexneri* infection that recapitulates human disease symptoms and features bacterial dissemination as an essential determinant of pathogenesis.

## Introduction

The intracellular pathogen *Shigella flexneri* is the causative agent of bacillary dysentery (bloody diarrhea) in humans^1^. In low-income countries, poor sanitation is responsible for ~270 million cases of shigellosis annually, resulting in more than 200,000 deaths^2^. In high-income countries, shigellosis is typically associated with travel to high-risk regions (Latin America, Asia, and Africa). The disease is associated with dramatic ulceration of the colonic mucosa and massive inflammation^3,4^. *Shigella flexneri* is transmitted via the fecal–oral route and is extremely contagious, with an attack rate above 90% with an infectious dose as low as 100–1000 bacteria per individual, as determined in human volunteer studies^5^. Until recently, infected patients were easily cured with antibiotic treatment. However, the isolation of multiple antimicrobial-resistant strains from infected patients is becoming alarmingly common worldwide^6^.

Seminal studies conducted in non-human primates have revealed that *S. flexneri* is an intracellular pathogen that resides in epithelial cells in the colon^7^. Tissue culture systems have been developed to model *S. flexneri* intracellular invasion^8^. The development of in vitro tissue culture systems was instrumental in our understanding of the molecular determinants supporting *S. flexneri* intracellular invasion. This led to the discovery that *S. flexneri* invasion relies on the presence of “the invasion plasmid”^9^ that harbors the 37 kb “entry region”^10^ encoding the type-3 secretion system (T3SS). The bacterial effector proteins, which are delivered into targeted host cells by the T3SS, manipulate various cellular processes, including the actin cytoskeleton, leading to the uptake of the bacteria by non-phagocytic cells, such as epithelial cells, into primary vacuoles^11^. Escape from primary vacuoles grants the pathogen access to the host cell cytosol. Cytosolic bacteria express a virulence factor, IcsA^12,13^, involved in the recruitment of the host cell actin polymerization machinery at the bacterial pole^14,15^. Actin polymerization propels the pathogen throughout the cytosol of infected cells and mediates the formation of membrane protrusions that project into adjacent cells at cell-cell contacts^16–19^. The formed protrusions resolve into secondary vacuoles, from which the pathogen escapes, thereby gaining access to the cytosol of adjacent cells and achieving cell-to-cell spread^20^.

There is a significant gap in knowledge as to how the molecular and cellular mechanisms supporting *S. flexneri* intracellular invasion and dissemination relate to pathogenesis. This is partly due to the lack of a small-animal model of bacillary dysentery. *Shigella flexneri* is a human-specific pathogen and the only known animals that display the symptoms observed in infected humans are non-human primates. Various small-animal models have been used in the past, including the mouse, the guinea pig, and the adult rabbit^21–26^. However, most of these models are not relevant to the site of *S. flexneri* infection in humans, that is, the colon. Moreover, none of these models recapitulate the hallmark of human shigellosis, that is, bloody diarrhea.

Here, we present an infant rabbit model of bacillary dysentery that recapitulates all the symptoms of human shigellosis, including mucosal ulceration, immune cell infiltration, and bloody diarrhea. The infant rabbit model shows that intracellular invasion of epithelial cells is required and sufficient for immune cell infiltration and vascular lesions. However, mucosal ulceration, bloody diarrhea, and weight loss are strictly contingent on the ability of the bacteria to spread from cell to cell.

## Results

### *Shigella flexneri*-mediated bloody diarrhea in infant rabbits

**Fig. 1:**
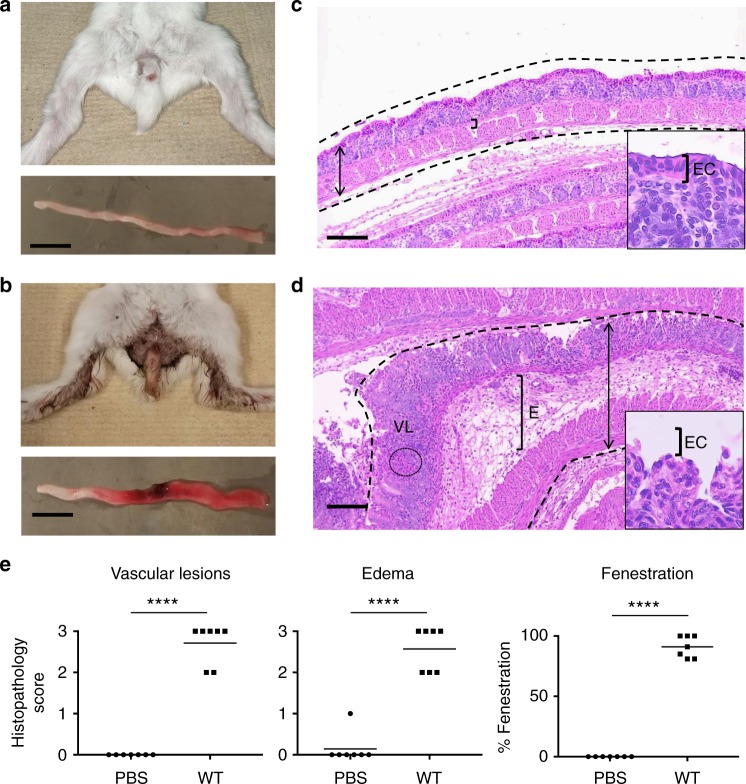
Symptoms and histopathology in infant rabbits infected with *Shigella flexneri*. **a**, **b** Representative images of animals (top) and colon (bottom) inoculated with phosphate-buffered saline (PBS) (**a**) or *S. flexneri* (**b**). Scale bar, 2 cm. **c**, **d** Representative images of hematoxylin- and eosin-stained colonic sections from animals inoculated with PBS (**c**) or *S. flexneri* (**d**). Dotted lines delineate the colon. Double arrows indicate the width of the colon section with massive swelling in **d**. Brackets indicate the width of connective tissues with edema (E). VL indicates vascular lesions with red blood cells. Insets: high magnification of epithelial cells (EC) with fenestration in **d**. Scale bars, 100 μm. **e** Histopathology scores of VLs, edema (E), and percentage of epithelial fenestration (EF). Statistical analysis, unpaired *t* test. *Shigella flexneri* (wild type (WT)) vs. mock (PBS): VL, ****P < 0.0001; E, ****P < 0.0001; EF, ****P < 0.0001. Source data are provided as a Source Data file

To establish a small-animal model of bacillary dysentery, we investigated the infant rabbit system because it has been successfully used for modeling infection with various human enteric pathogens that reside either in the small intestine or in the colon, such as *Vibrio cholerae* and *Enterohemorrhagic Escherichia coli*, respectively^27,28^. We succeeded in infecting infant rabbits by oral route, but the infection outcome was inconsistent. Reasoning that *S. flexneri* may experience a bottleneck in the stomach or the small intestine of infant rabbits, we developed a model of infection by rectal route, thereby delivering *S. flexneri* at its natural site of infection, the distal colon (Supplementary Fig. 1). In this model, all challenged animals developed bloody diarrhea within 24 h post inoculation (pi) (Table 1), while mock-treated rabbits did not display any signs of disease (Fig. 1a, b). On average, 50% of the infected animals lost more than 15% of their initial body weight within 3 days and were euthanized according to our animal protocol.

**Table 1:** Bacillary dysentery in infant rabbits inoculated with wild-type and mutant *S. flexneri*

Strain	2457T	2457T *ΔmxiG* (invasion)	2457T *ΔicsA* (spread)
Total number of animals	43	28	31
Disease category^a^			
Severe bloody diarrhea	36 (84%)	0	0
Mild bloody diarrhea	7 (16%)	0	0
Watery discharge^b^	0	0	8 (26%)
No symptoms	0	28 (100%)	23 (74%)
% Bloody diarrhea	100%	0%	0%

^a^Total number of animals in each category and percentage with respect to total number of animals

^b^Hind legs with tarnished fur, but no watery or bloody diarrhea

### *Shigella flexneri*-mediated mucosal ulceration in infant rabbits

Upon dissection, all animals challenged for 24 h with wild-type *S. flexneri* displayed a distal colon that appeared swollen and showed redness suggestive of hemorrhage that extended from the site of inoculum delivery to the rectum (Fig. 1a, b and Supplementary Fig. 1). The proximal colon upstream the site of inoculation appeared thin and white, similar to the appearance of the whole distal colon in mock-treated animals (Fig. 1a, b and Supplementary Fig. 1). Histological examination revealed expected structural features of the colonic mucosa in mock-treated animals, including crypts and a continuous lining of epithelial cells separating the lumen from the underlying sub-mucosa, constituted of connective tissues and muscle layers (Fig. 1c and Supplementary Fig. 1). In challenged animals, in agreement with the bloody diarrhea symptom and the redness of the colon (Fig. 1b), we observed numerous red blood cells in the mucosa (Supplementary Fig. 1), indicative of vascular lesions (VLs) (Fig. 1d). The tissue displayed evident signs of connective tissue and muscle edema (Fig. 1d, e and Supplementary Fig. 1). Strikingly, massive damage was inflicted on the epithelial cells facing the lumen, resulting in a nearly complete fenestration of the epithelial layer, exposing the sub-mucosa to the lumen, but leaving the base of the crypts intact (Fig. 1b–d, insets, and Supplementary Fig. 1). To quantify these observations, we used a scoring system grading the severity of the observed phenotypes (Supplementary Table 1). The approach showed that infant rabbits infected with wild-type *S. flexneri* experienced bloody diarrhea associated with statistically significant VLs (*P* < 0.0001), edema (*P* < 0.0001), and epithelial fenestration (EF) (*P* < 0.0001) (Fig. 1e).

### *Shigella flexneri*-mediated pro-inflammatory immune responses

**Fig. 2:**
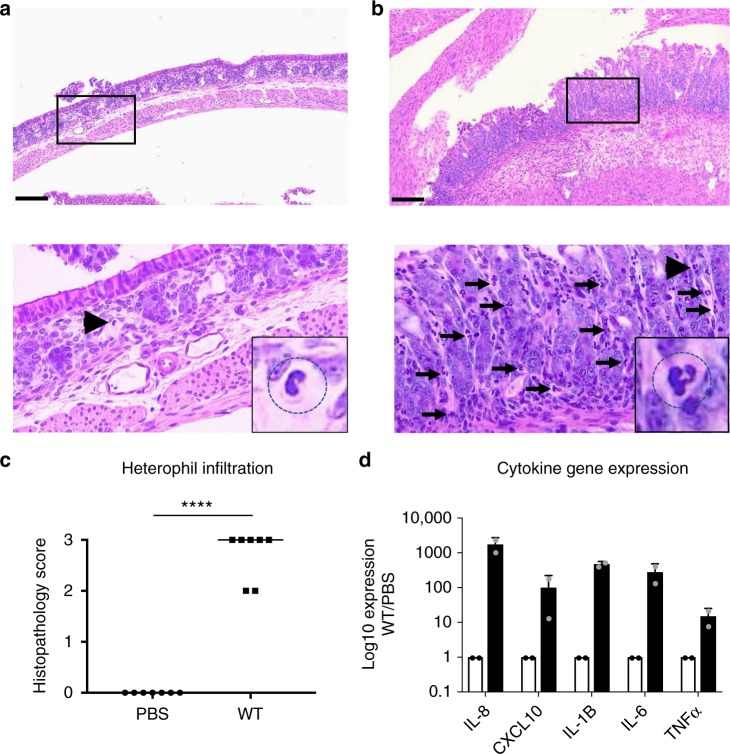
Pro-inflammatory immune responses in the colon of infant rabbits infected with *Shigella flexneri*. **a**, **b** Representative images of hematoxylin- and eosin-stained colonic sections from animals inoculated with phosphate-buffered saline (PBS) (**a**) or *S. flexneri* (**b**). Scale bars, 100 μm. Top, ×10 images; bottom, ×40 images of the boxed area in top images. Arrows and arrowheads indicate heterophils. Insets, zoom-in on the heterophil indicated by arrowheads. **c** Graph showing histopathology score of heterophil infiltration. Statistical analysis, unpaired Student’s *t* test. Wild-type (WT) vs. mock, ****P < 0.0001. **d** Chemokine (interleukin-8 (IL-8) and C-X-C motif chemokine 10 (CXCL10)) and cytokine (IL-1β, IL-6, and tumor necrosis factor-α (TNFα)) gene induction (black bars) in the colon 8 h post inoculation (pi) with *S. flexneri* with respect to mock-treated animals (PBS, white bars). Overlay dot plots represent individual data points. Error bars represent standard deviation of the mean. Source data are provided as a Source Data file

Human shigellosis is characterized by a massive infiltration of polymorphonuclear leukocytes (PMNs), including neutrophils. In rabbits, PMNs are referred to as heterophils. In infant rabbits infected with *S. flexneri*, the mucosa and sub-mucosa appeared densely populated by heterophils 24 h pi (Fig. 2a–c), indicating a massive infiltration of immune cells in response to infection. In addition to PMNs, we also observed infiltration of monocytes (Supplementary Fig. 2). To further characterize the infant rabbit immune response to *S. flexneri* infection, we designed rabbit-specific primers for amplification of conserved cytokine and chemokine genes (Supplementary Table 2). Gene expression analysis showed a massive pro-inflammatory response in the colon of infected infant rabbits as determined by transcriptional expression of chemokines such as interleukin-8 (IL-8) and C-X-C motif chemokine 10 (CXCL10), and cytokines such as IL-1β, IL-6, and tumor necrosis factor-α (TNFα) (Fig. 2d). Collectively, these results demonstrate that in infant rabbits, *S. flexneri* infection consistently leads to pro-inflammatory responses including the expression of chemokines, such as IL-8, that correlates with the massive infiltration of immune cells, including heterophils.

### T3SS-dependent invasion of epithelial cells in the colon

**Fig. 3:**
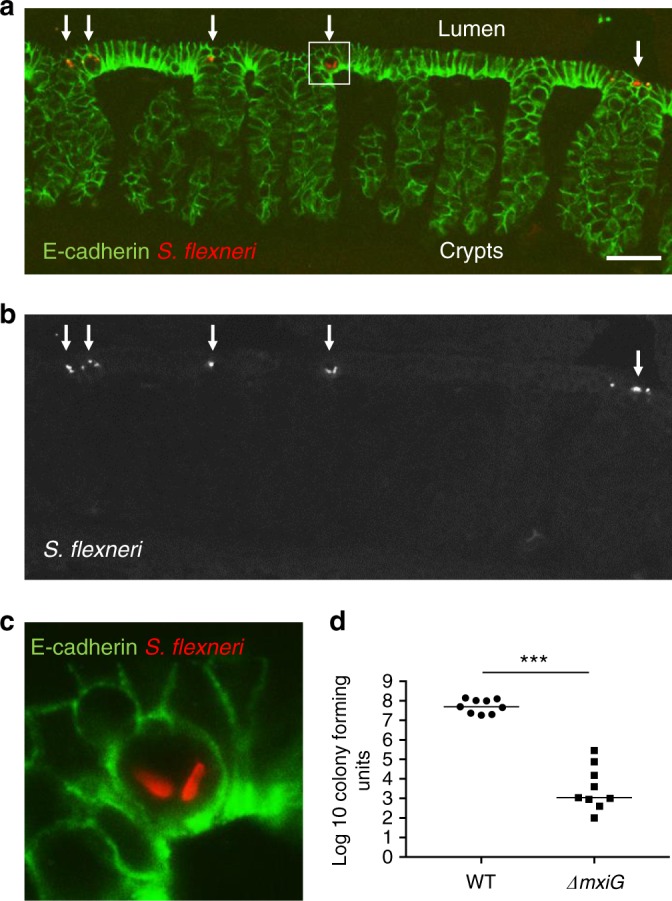
*Shigella flexneri* invades epithelia cells in a T3SS-dependent manner. **a**, **b** Representative images of colonic sections immuno-stained for E-cadherin and *S. flexneri*. **a** Merge: E-cadherin, green; *S. flexneri*, red. **b** *S. flexneri* only. Arrows indicate intracellular *S. flexneri*. Scale bars, 50 μm. **c** Zoom-in on the boxed area in **a**. **d** Graph showing counts of colony-forming units in the distal colon of animals infected with *S. flexneri* (wild-type (WT)) or the T3SS mutant (*ΔmxiG*). Statistical analysis, unpaired *t* test. *ΔmxiG* vs. WT, ***P < 0.0005. Source data are provided as a Source Data file

Seminal electron microscopy studies conducted in non-human primates have first uncovered the notion that *S. flexneri* is an intracellular pathogen that resides within epithelial cells in the colon^7^. In infant rabbits, *S. flexneri* was found in association with colonic E-cadherin-positive epithelial cells 2 h pi (Fig. 3a, b). The vast majority of *S. flexneri* was associated with epithelial cells located at the top of the crypts and rarely with luminal epithelial cells located in between crypts (Fig. 3a, b). High-magnification imaging of thin sections unambiguously demonstrated that *S. flexneri* resided within epithelial cells, showing that similar to observations in non-human primates, *S. flexneri* resides within epithelial cells in the colon of infant rabbits (Fig. 3c). In human tissue culture systems, *S. flexneri* relies on its T3SS to invade non-phagocytic cells^11^. To determine the role of the T3SS in *S. flexneri* infection in infant rabbits, we used a mutant defective in the expression of *mxiG*^29^, which encodes a structural component of the T3SS. We have previously shown that the *ΔmxiG* mutant is unable to invade epithelial cells, such as human colonic HT-29 cells^29^. As expected, wild-type *S. flexneri* and the *ΔmxiG* mutant were readily detectable 2 h pi in the colon of infant rabbits, using standard assays such as colony-forming unit (CFU) determination. However, after dissection and immuno-staining procedures, the *ΔmxiG* mutant was never observed within E-cadherin-positive epithelial cells, or in association with the colonic tissue altogether (Supplementary Fig. 3). These results indicate that in the colon of infant rabbits, *S. flexneri* invades epithelial cells through a mechanism that requires the integrity of the T3SS.

Although the *ΔmxiG* was unable to invade epithelial cells, we tested whether it could nonetheless colonize the colon, perhaps extracellularly. To this end, we conducted a plating assay to determine if we could recover CFUs from the colon 24 h pi. The approach revealed that we could recover 10–100 million CFUs from animals infected with wild-type *S. flexneri*. By contrast, we recovered ~1000 CFUs in average from animals infected with the *ΔmxiG* mutant (Fig. 3d). These results indicate that, in addition to the lack of intracellular invasion, the T3SS-defective strain cannot colonize the colon of infant rabbits.

### Role of T3SS-dependent invasion in pathogenesis

**Fig. 4:**
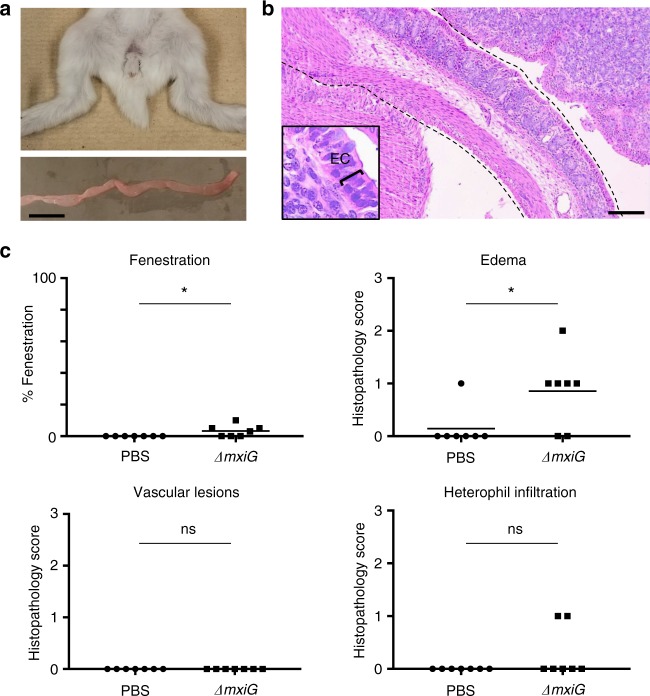
Symptoms and histopathology in absence of intracellular invasion. **a** Representative images of animals (top) and colon (bottom) inoculated with the Δ*mixG* mutant. Scale bar, 2 cm. **b** Representative image of hematoxylin- and eosin-stained colonic sections from animals inoculated with the *ΔT3SS* mutant. Dotted lines delineate the colon. Inset: high-magnification of epithelial cells (ECs). Scale bars, 100 μm. **c** Percentage of epithelial fenestration (EF), and histopthology scores of edema (E), vascular lesions (VLs), and heterophil infiltration (HI). Statistical analysis, unpaired *t* test. *ΔmxiG* vs. mock (phosphate-buffered saline (PBS)): EF, *P < 0.05; E, *P < 0.05; VL, ns; HI, ns (not significant). Source data are provided as a Source Data file

We next determined whether the *ΔmxiG* mutant, though not able to colonize the colon efficiently, displayed transient pathogenic properties. Animals infected with the *ΔmxiG* mutant did not display any signs of diarrhea, and their colon appeared healthy upon dissection (Table 1 and Fig. 4a, b). Histological evaluation of pathology revealed minor but significant defects 24 h pi, including EF and edema (Fig. 4c). VLs were not observed and the observed immune cell infiltration in infected animals was not significantly different from control animals (Fig. 4c). These experiments suggest that, in addition to the T3SS and secreted effector proteins, *S. flexneri* may display additional virulence factors that challenge the intestinal mucosa. However, these mild defects did not lead to any discernable symptoms of disease, such as diarrhea (Table 1). Collectively, these results indicate that in infant rabbits, *S. flexneri* is an intracellular pathogen that relies on the integrity of the T3SS to cause significant disease.

### Bacterial spread from cell to cell in the colon

**Fig. 5:**
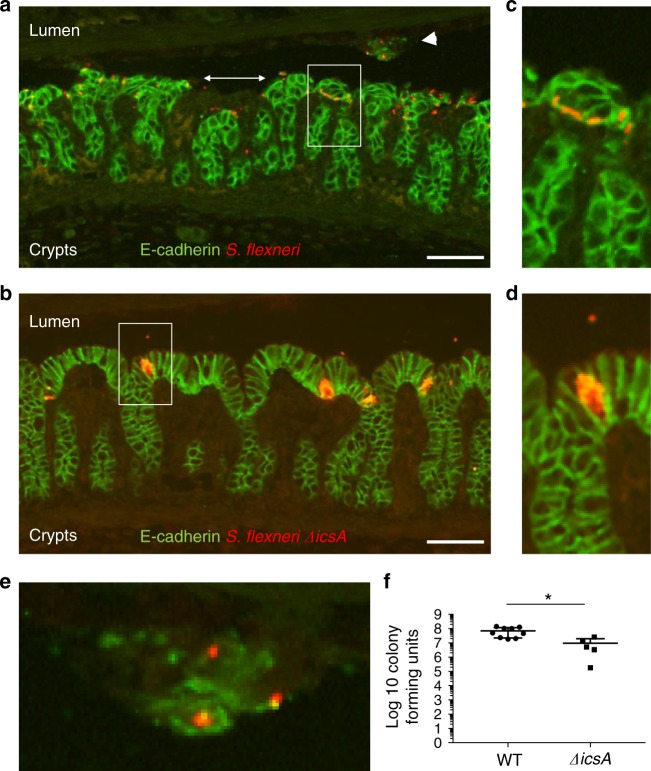
*Shigella flexneri* spreads from cell to cell in an IcsA-dependent manner. **a**, **b** Representative images of colon sections infected with *S. flexneri* (**a**) and the *ΔicsA* mutant (**b**). Scale bars, 50 μm. E-cadherin, green; *S. flexneri*, red. **c**, **d** Zoom-in on the boxed area in **a**, **b**, respectively. **e** Zoom-in on the area indicated by the arrowhead in **a**. **f** Graph showing counts of colony-forming units in the distal colon of animals infected with *S. flexneri* (wild-type (WT)) or the *ΔicsA* mutant. Statistical analysis: unpaired *t* test. *ΔicsA* vs. *S. flexneri* (WT), *P < 0.05. Source data are provided as a Source Data file

In human cell culture monolayers, *S. flexneri* invades a few isolated cells, displays IcsA-dependent actin-based motility in the cytosol of infected cells, and readily spreads to adjacent cells (Supplementary Fig. 4). As bacteria multiply and disseminate, *S. flexneri* infection appears as groups of adjacent cells containing intracellular bacteria, referred to as infection foci (Supplementary Fig. 4). This is in contrast with the *ΔicsA* mutant, which is as invasive as wild type, but does not spread from cell to cell, and thus grows as micro-colonies in primarily infected cells (Supplementary Fig. 4). In the colon of infant rabbits, wild-type bacteria and the *ΔicsA* mutant were found within isolated epithelial cells 2 h pi (Supplementary Fig. 5). Later on, 8 h pi, wild-type *S. flexneri* was observed in groups of adjacent cells (Fig. 5a–c), showing that bacteria had multiplied and spread from cell to cell. By contrast, the *ΔicsA* mutant was found in isolated cells 8 h pi, where it had grown as micro-colonies (Fig. 5b–d). Thus, similar to the situation observed in human cell culture monolayers, the *ΔicsA* mutant failed to spread from cell to cell in the colonic epithelium of infant rabbits. The lining of the epithelium appeared intact in animals infected with the *ΔicsA* mutant (Fig. 5b), which was in stark contrast with the obvious fenestration of the colonic epithelium in animals infected with wild-type *S. flexneri* (Fig. 5a, double arrow). Accordingly, we observed groups of infected epithelial cells in the lumen that apparently detached from the rest of the epithelium (Fig. 5a, arrowhead and Fig. 5e). In agreement with these observations, and in contrast with the T3SS-defective mutant (Fig. 3d, *ΔmxiG*), the *ΔicsA* mutant was able to colonize the colon, as tested by CFU determination (Fig. 5f). Collectively, these results indicate that *S. flexneri* spreads from cell to cell in an IcsA-dependent manner in the colon of infant rabbits. The results also suggest that the dissemination process contributes very early on (8 h pi) to epithelial cell fenestration (see below).

### Role of cell-to-cell spread in pathogenesis

**Fig. 6:**
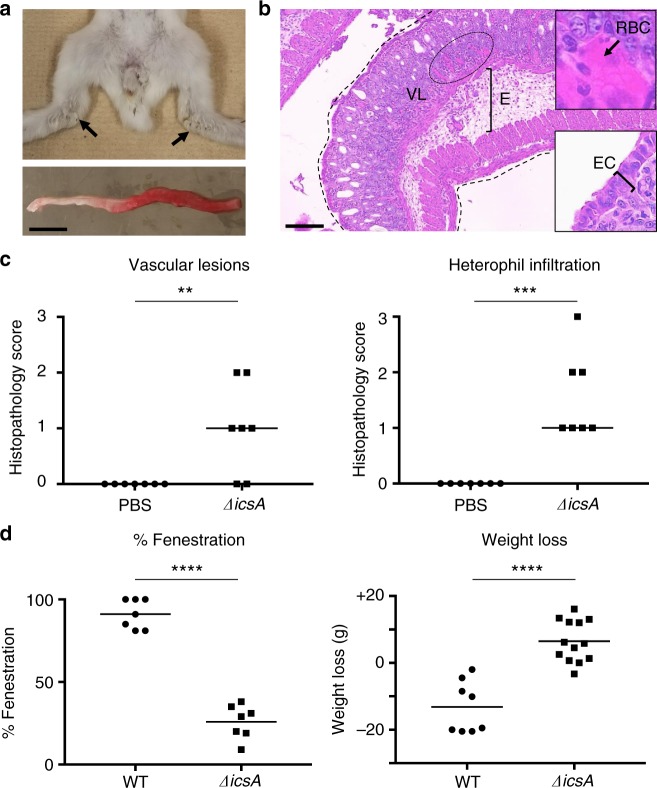
Symptoms and histopathology in absence of cell-to-cell spread. **a** Representative images of animals (top) and colon (bottom) inoculated with the *ΔicsA* mutant. Arrows indicate tarnished fur. Scale bar, 2 cm. **b** Representative image of hematoxylin- and eosin-stained colonic sections from animals inoculated with the *ΔicsA* mutant. Dotted lines delineate the colon. E, edema; VLs, vascular lesions. Insets: (top) high magnification of red blood cells (RBCs) indicative of VLs and (bottom) high magnification of epithelial cells (ECs). Scale bar, 100 μm. **c** Histopathology scores of VLs) and heterophil infiltration (HI). Statistical analysis: unpaired Student’s *t* test. *ΔicsA* vs. mock (phosphate-buffered saline (PBS): VLs, **P < 0.01; ***HI, *P* < 0.05. **d** Graphs showing the percentage of epithelial fenestration (EF) and weight loss (WL). Statistical analysis, unpaired *t* test. *ΔicsA* vs. *S. flexneri* (wild type (WT)): EF, ****P < 0.0001; WL, ****P < 0.0001. Source data are provided as a Source Data file

We next determined the importance of the dissemination process in pathogenesis. Remarkably, animals infected with the *ΔicsA* mutant did not display bloody diarrhea (Fig. 6a and Table 1). A few animals (~25%) experienced a mild watery discharge that tarnished the fur of their hind legs (Fig. 6a, arrows, and Table 1). This symptom did not progress beyond 24 h pi and never evolved into watery or bloody diarrhea. Surprisingly, given the complete absence of bloody diarrhea, the colon of animals infected with the *ΔicsA* mutant appeared bloody (Fig. 6a). Histological analyses confirmed the presence of VLs as the mucosa displayed numerous red blood cells (Fig. 6b, VLs and inset, red blood cells; and Fig. 6c, VLs). We also observed significant edema (Supplementary Fig. 6a). Chemokine and cytokine genes were highly induced (Supplementary Fig. 6b) and immune cells infiltrated the tissue (Fig. 6c, heterophil infiltration, and Supplementary Fig. 2c, monocyte infiltration). A major difference between the *ΔicsA* mutant and wild-type *S. flexneri* was the relative integrity of the colonic mucosa (compare Figs. 1d and 6b, and corresponding insets). While infection with wild-type *S. flexneri* led to almost complete EF 24 h pi, the mucosal layer appeared largely intact in animal infected with the *ΔicsA* mutant (Fig. 6d, fenestration, IcsA vs. WT, *P* < 0.0001). We also evaluated the impact of infection on weight loss 3 days pi. While the animals infected with the *ΔicsA* mutant gained weight, animals infected with wild-type *S. flexneri* experienced a dramatic weight loss (Fig. 6d, weight loss, *P* < 0.0001). Collectively, these results indicate that *S. flexneri* intracellular invasion is necessary and sufficient for immune cell infiltration and VLs. However, EF, bloody diarrhea, and weight loss are contingent on the ability of the bacteria to display intracellular spread from cell to cell.

## Discussion

*Shigella flexneri* is an intracellular pathogen that causes bloody diarrhea in humans and non-human primates. Various small-animal models including the mouse have been tested by oral infection and did not lead to productive infection^21^. Non-intestinal models of inflammation in response to *S. flexneri* challenge have been used, including the guinea pig eye and the mouse lung^23,25^. The mechanisms supporting intestinal inflammation have been explored using the ileal loop of adult rabbits as a model system^30–32^. It is noteworthy that none of these animal models are relevant to the site of *S. flexneri* infection in humans, that is, the colon, and recapitulate the hallmark of human shigellosis, that is, bloody diarrhea. Recently, a guinea pig model has been tested by rectal inoculation, which led to liquid stools and weight loss 24 h pi^24^. Although this model displayed expected innate and adaptive responses to infection, critical characteristics of *S. flexneri* infection, including intracellular invasion and bacterial dissemination, were not investigated. Here, we have developed a reliable model of bacillary dysentery in infant rabbits. Similar to the symptoms observed in infected human individuals, infected infant rabbits experienced severe ulceration of the intestinal mucosa and bloody diarrhea. Moreover, infection with bacterial mutants allowed us to determine the respective roles of intracellular invasion and dissemination in pathogenesis. Our results demonstrate that cell-to-cell spread is required for ulceration of the mucosa and bloody diarrhea, showing that a spreading defective mutant is attenuated in infant rabbits. These results are in agreement with previous studies showing that a spreading defective mutant was attenuated in non-human primates^33^. The infant rabbit model therefore constitutes the first small-animal model of bacillary dysentery that recapitulates all of the symptoms observed in non-human primates, and in infected patients.

We used the infant rabbit model to characterize the mechanisms supporting intracellular infection in vivo. Our work demonstrates that *S. flexneri* invasion of non-phagocytic cells relies on the integrity of the T3SS in the colon of infant rabbits. Importantly, our results show that *S. flexneri* directly invades epithelial cells in the colon. This is important because previous studies have proposed the now widespread notion that similar to various intestinal pathogens, *S. flexneri* targets M cells for invasion of the intestinal tract^34^. It is however noteworthy that this notion was established using the ileal loop of adult rabbits as a model system. While it is unclear whether *S. flexneri* invades the mucosa of the small intestine in humans, our results suggest that colonic infection is primarily supported by invasion of epithelial cells in a T3SS-dependent manner.

Human shigellosis is characterized by a massive ulceration of the colonic mucosa^3,4^. It is widely accepted that the immune system, mainly neutrophils in humans, is largely responsible for the damage inflicted on the mucosa during *S. flexneri* infection. This notion was first established through studies conducted in the ileal loop of adult rabbits, in which depleting PMNs, or blocking IL-1β signaling, significantly limited the extent of the observed damage^30,31^. However, the role of inflammation in shigellosis is complex, as similar studies relying on blocking IL-8 signaling showed exacerbated bacterial growth, suggesting in fact a protective role for immune cell infiltration in *S. flexneri* infection^32^. Similarly, neutrophil depletion studies using human colon xenografts in SCID mice did not ameliorate mucosal ulceration and resulted in exacerbated bacterial growth^35^. In the infant rabbit model, we observed a marked immune cell infiltration in animals infected with wild-type bacteria or the *ΔicsA* mutant, but pro-inflammatory responses were much milder or absent in animals infected with the T3SS-deficient mutant (*ΔmxiG*). These observations indicate that immune cell infiltration, in response to chemokine production, is primarily a response to intracellular infection of epithelial cells. Intracellular infection of epithelial cells is also required for VLs, through an unclear mechanism, that may or may not be an indirect consequence of immune cell infiltration. In spite of immune cell infiltration, animals infected with the *ΔicsA* mutant did not experience the massive EF observed in animals infected with wild-type bacteria. This striking difference in the response of the epithelium suggests that the damage inflicted on the epithelium is not directly due to immune cell infiltration. Instead, our results suggest a critical role for cell-to-cell spread in mucosal ulceration through massive EF, through a mechanism that remains to be elucidated.

**Fig. 7:**
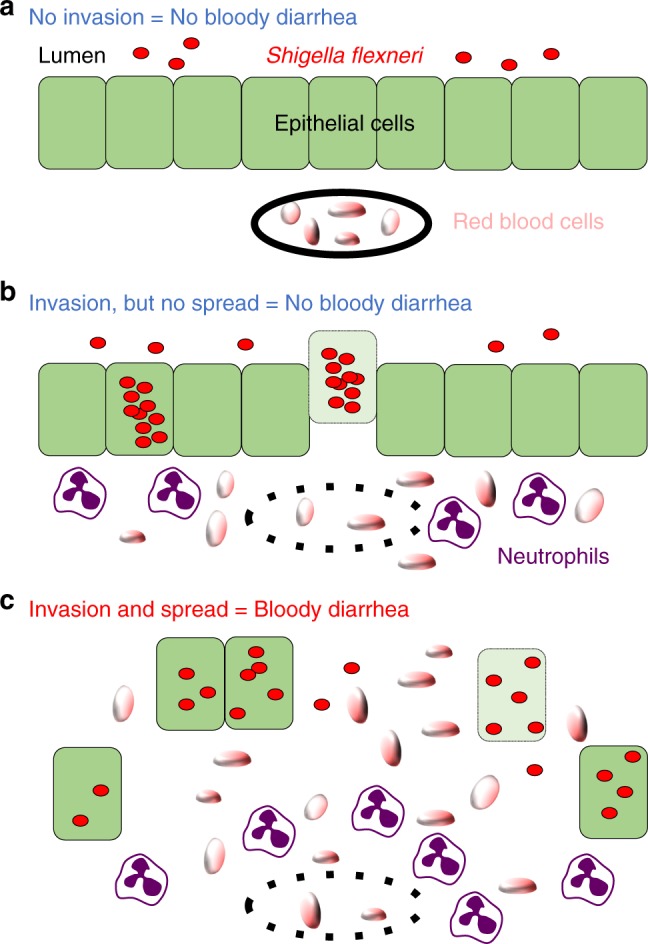
Model of bacillary dysentery. **a** In the absence of intracellular invasion (i.e. *ΔT3SS* mutant) of epithelial cells (green), *S. flexneri* (red) is present in the lumen, and red blood cells (tangerine) are confined to blood vessels (black line). Infected animals do not display any symptoms (no bloody diarrhea). **b** In the absence of cell-to-cell spread (i.e., *ΔicsA* mutant), *S. flexneri* grows as micro-colony in primarily infected cells, but does not get access to adjacent cells. Epithelial cell production of signaling molecules (not shown) and vascular lesions (doted black line) lead to the infiltration of immune cells (neutrophils, purple) and red blood cells, respectively. In the absence of cell-to-cell spread, infected epithelial cells may undergo cell death (pale green) and be extruded from the epithelium. Fenestration of the epithelial layer is minimal and animals do not display any symptoms (no bloody diarrhea). **c** Wild-type *S. flexneri* invades epithelial cells and spreads from cell to cell. Similar to **b**, intracellular invasion leads to immune cell infiltration and vascular lesions. In addition, cell-to-cell spread leads to massive epithelial cell fenestration, which correlates with bacillary dysentery symptoms (bloody diarrhea)

Based on the results presented here, we propose the following model of bacillary dysentery (Fig. 7). In the colonic mucosa, *S. flexneri* relies on the T3SS for invading epithelial cells. The presence of intracellular bacteria leads to signaling events that mediate the recruitment of immune cells. VLs may be the consequence of immune cell infiltration. It is possible, however, that signaling events from infected epithelial cells contribute to VLs, independent of immune cell infiltration. In addition to intracellular invasion, bacterial spread from cell to cell leads to near complete fenestration of the epithelium, and bloody diarrhea. We speculate that EF is critical for the release of red blood cells into the lumen, and hence supports bloody diarrhea. How bacterial spread from cell to cell leads to EF is unknown. The infant rabbit model thus provides a unique framework to uncover the bacterial factors, as well as the host cell processes, supporting bacterial dissemination and how these mechanisms relate to *S. flexneri* pathogenesis in vivo.

## Methods

### Cell lines and bacterial strains

HT-29 cells (ATCC HTB-38) and HT-29 cells stably expressing yellow fluorescent protein membrane markers were cultured at 37 °C with 5% CO_2_ in McCoy’s 5A medium (Gibco) supplemented with 10% heat-inactive fetal bovine serum (Invitrogen). The wild-type *S. flexneri* strain used in this study was serotype 2a 2457T (a gift from Dr. Marcia Goldberg) and was cultured in Luria-Bertani (LB) or tryptic soy broth (TSB) media at 37 °C. The T3SS-deficient 2457T strain lacks the *mxiG* gene^29^, which encodes a structural component of the T3SS. The *ΔicsA* 2457T strain was generated by replacing the *icsA* coding region with the coding region of a chloramphenicol resistance cassette.

### Animal procedures

Pregnant female New Zealand White rabbits were obtained from a commercial breeding company (Charles River). Newborns were isolated after birth and kept in a 30 °C incubator for the remaining duration of the experiment. At the time of feeding, the does were dosed intramuscularly with 0.5 ml of acetylpromazine and 0.3 ml of oxytocin, infant rabbits were fed on the tranquilized does, and then put back in the incubator. For infecting 10–15-day-old infant rabbits, *S. flexneri* was grown overnight at 37 °C on a rotating wheel in 5 ml TSB per animal. The bacterial culture was pelleted and resuspended in 200 μl PBS prior to inoculation. Infant rabbits were anesthetized in a chamber with 5% isoflurane in 5 l/min oxygen before rectal inoculation with 200 μl bacterial suspension (~10^9^ CFUs per animal) using feeding tubes (Instech Labs, FTP-20–38). Post inoculation, the infant rabbits were weighed daily pre-feeding and post feeding and observed twice daily for clinical signs of illness.

All experiments described in this study were reviewed and approved by the University of Virginia Institutional Biosafety Committee and the Institutional Animal Care and Use Committee. Care of the does and infant rabbits adhered to standard operating procedures developed in coordination with the veterinary and animal care staff of the Center for Comparative Medicine at the University of Virginia.

### Histology

At the appropriate time point, infant rabbits were euthanized by CO_2_ inhalation and the distal colon (10 cm) was harvested. The samples were rinsed in PBS and flushed with modified Bouin’s fixative for paraffin sections, or processed directly for frozen sections. Samples were cut open longitudinally and displayed in cassettes as swiss-rolls. Cassettes were immersed in neutral-buffered formalin or 4% formaldehyde for paraffin and frozen sections, respectively. For paraffin slides, the tissue was preserved in 70% EtOH before loading onto a tissue processor for dehydration and paraffin infiltration. After manual embedding into a paraffin block, paraffin sections were cut at 5 μm on a Leica microtome. For frozen sections, samples were cryopreserved in 30% sucrose, and then embedded in OCT inside the cryostat at −20 °C. Cryo-sections were cut at 7 μm and slides stored at −80 °C.

### Histopathology analysis

Paraffin sections of colonic tissue were stained with hematoxylin and eosin. Representative images were taken using a Leica DM750 microscope and a Leica ICC50W camera. Scoring of blinded tissue sections was performed by a veterinary pathologist based on the criteria listed in Supplementary Table 1. The scoring system evaluates VLs, sub-mucosal edema, EF, heterophil infiltration, and monocyte infiltration. Percentage of EF for each individual sample was calculated using the Leica LAS X imaging software: EF = (length of colonic epithelium with signs of fenestration/total length of colonic epithelium) × 100.

### Colonic RNA extraction

At the appropriate time point, the colon was removed from euthanized animals and placed directly in 6 ml of TRIzol (Thermo Fisher Scientific, 15596026) in a 50 ml falcon tube. Samples were homogenized in TRIzol using the Tissue Master 125 Lab Homogenizer with a 7 mm probe (Omni International, TM125-115). One milliliter of the TRIzol homogenate was placed into a 1.5 ml microfuge tube containing 200 μl of chloroform. Samples were vigorously shaken, incubated at room temperature for 2–3 min, and centrifuged at 12,000 rcf for 10 min. Five hundred microliters of the aqueous (top) phase was placed into a 2.0 ml microfuge tube that contained 500 μl isopropanol and 20 ng/μl glycogen, and mixed by inversion. Samples were stored as a mixture with isopropanol at −20 °C until further processing. The RNA samples in isopropanol were precipitated and washed with 75% ethanol following the TRIzol Reagent User Guide. Samples were resuspended in 100 μl of RNase-free water and stored in 10 μl aliquots at −80 °C.

### Real-time PCR analysis of immune gene expression

After RNA extraction, each 10 μl RNA sample was diluted with 35 μl of RNase-free water and treated with DNase (2 × 1 μl DNase for 1 h) following the TURBO DNA-free kit protocol (Thermo Fisher Scientific, AM1907). Forty five microliters of RNA was retrieved per sample. Complementary DNA (cDNA) was synthesized using SuperScript II reverse transcriptase (Thermo Fisher Scientific, 18064-022), following the manufacturer’s protocol. Samples were primed using random primers (Thermo Fisher Scientific: 48190011). cDNA samples were diluted 1 to 5 in water and stored at −20 °C. Each quantitative PCR reaction consisted of the following: 10 μl Probe master mix (New England Biolabs, M3004), 1 µl cDNA, 0.2 μl probe (Roche Universal Probe Library), 0.2 μl primer pair (each primer at 20 nM), and 8.6 μl water for a total reaction volume of 20 μl. The cycling conditions on a LightCycler 96 Instrument (Roche: 05815916001) were as follows: pre-incubation (1 cycle) 5 min at 95 °C; Amplification (45 cycles) 10 s at 95 °C, 20 s at 60 °C, 1 s at 72 °C; cooling (1 cycle) 10 s at 40 °C. Measurements were taken during 1 s 72 °C step. Cq values were derived using the LightCycler 96 software and fold changes were calculated using an uninfected sample and GAPDH (glyceraldehyde 3-phosphate dehydrogenase) as a housekeeping gene for normalization.

### Plating assay and CFU determination

At the appropriate time point, the colon was removed from euthanized animals and placed directly in 10 ml PBS in a 50 ml falcon tube. Samples were homogenized using the Tissue Master 125 Lab Homogenizer with 7 mm probe (Omni International, TM125-115) until no chunks of tissue were visible. The homogenizer was rinsed in the following sequential solutions: water, 70% ethanol, water. Adhered tissue was removed and the probe placed in 70% ethanol. The probe was rinsed one final time in water before homogenizing the next sample. One in 10 serial dilutions of the samples were made from 10^−1^ to 10^−5^ and 100 μl of the original sample and its dilutions were plated on selective LB Congo red agar plates and placed at 37 °C. CFUs were counted the following day.

### Immunofluorescence microscopy

Paraffin sections were re-hydrated in the following sequence: xylene, 100% ethanol, 95% ethanol, 70% ethanol, and water. Antigen retrieval was performed at 95–100 °C for 20 min using a pre-heated citric acid-based buffer (Vector Laboratories, H-3300). Slides were cooled in the buffer solution for 1 h at room temperature and washed in PBS 2× for 5 min each. Slides were permeabilized in 0.1% Triton for 10 min at room temperature and washed in PBS for 5 min. Samples were blocked in 5% bovine serum albumin and 2% normal goat serum in PBS for 1 h at room temperature. Primary antibodies were diluted 1:100 in blocking buffer (E-cadherin, BD Biosciences 610181; *Shigella* sp., ViroStat 0901) and incubated overnight at 4 °C. Secondary antibodies (goat anti-mouse Alexa Fluor 514, Thermo Fisher Scientific, A-31555; goat anti-rabbit Pacific Blue: Thermo Fisher Scientific, P10994) were diluted 1:500 and 1:200, respectively, in blocking buffer and incubated for 2 h at room temperature. Coverslips were mounted using ProLong Gold Antifade Mountant (Thermo Fisher Scientific, P36930). Slides were imaged using a Nikon TE2000 microscope equipped for automated multi-color imaging including motorized stage and filter wheels, a Hamamatsu Orca ER Digital CCD Camera and piezo-driven ×10 and ×60 objectives. The corresponding images were processed with the MetaMorph software (Molecular Devices, Inc.).

### Statistical analysis

Statistical analyses of histopathology scores were conducted with the Prism software. Unpaired *t* tests or one-way analysis of variance were reported as follows: *P < 0.05; **P < 0.01; ***P < 0.001, and ****P < 0.0001. To limit potential litter-specific differences, at least three independent litters were used for all the conditions tested, and the corresponding histopathology scores were pooled for statistical analyses.

### Reporting summary

Further information on research design is available in the Nature Research Reporting Summary linked to this article.

## Supplementary information

Supplementary Information

Reporting Summary

Source Data

## Data Availability

All data generated or analyzed during this study are included in this published article (and its Supplementary Information files). The source data underlying Figs. 1e, 2c, d, 3d, 4c, 5f and 6c, d and Supplementary Figs. 2c and 6b are provided as a Source Data file.

## References

[1] Musher DM, Musher BL (2004). Contagious acute gastrointestinal infections. N. Engl. J. Med..

[2] Khalil Ibrahim A, Troeger Christopher, Blacker Brigette F, Rao Puja C, Brown Alexandria, Atherly Deborah E, Brewer Thomas G, Engmann Cyril M, Houpt Eric R, Kang Gagandeep, Kotloff Karen L, Levine Myron M, Luby Stephen P, MacLennan Calman A, Pan William K, Pavlinac Patricia B, Platts-Mills James A, Qadri Firdausi, Riddle Mark S, Ryan Edward T, Shoultz David A, Steele A Duncan, Walson Judd L, Sanders John W, Mokdad Ali H, Murray Christopher J L, Hay Simon I, Reiner Robert C (2018). Morbidity and mortality due to shigella and enterotoxigenic Escherichia coli diarrhoea: the Global Burden of Disease Study 1990–2016. The Lancet Infectious Diseases.

[3] Anand BS (1986). Rectal histology in acute bacillary dysentery. Gastroenterology.

[4] Mathan MM, Mathan VI (1991). Morphology of rectal mucosa of patients with shigellosis. Rev. Infect. Dis..

[5] DuPont HL, Hornick RB, Dawkins AT, Snyder MJ, Formal SB (1969). The response of man to virulent *Shigella flexneri* 2a. J. Infect. Dis..

[6] Puzari M, Sharma M, Chetia P (2018). Emergence of antibiotic resistant *Shigella* species: a matter of concern. J. Infect. Public Health.

[7] Takeuchi A, Formal SB, Sprinz H (1968). Experimental acute colitis in the Rhesus monkey following peroral infection with *Shigella flexneri*. An electron microscope study. Am. J. Pathol..

[8] Labrec EH, Schneider H, Magnani TJ, Formal SB (1964). Epithelial cell penetration as an essential step in the pathogenesis of bacillary dysentery. J. Bacteriol..

[9] Sansonetti PJ, Kopecko DJ, Formal SB (1982). Involvement of a plasmid in the invasive ability of *Shigella flexneri*. Infect. Immun..

[10] Maurelli AT, Baudry B, d’Hauteville H, Hale TL, Sansonetti PJ (1985). Cloning of plasmid DNA sequences involved in invasion of HeLa cells by *Shigella flexneri*. Infect. Immun..

[11] Carayol N, Tran Van Nhieu G (2013). The inside story of *Shigella* invasion of intestinal epithelial cells. Cold Spring Harb. Perspect. Med..

[12] Bernardini ML, Mounier J, d’Hauteville H, Coquis-Rondon M, Sansonetti PJ (1989). Identification of *icsA*, a plasmid locus of *Shigella flexneri* that governs bacterial intra- and intercellular spread through interaction with F-actin. Proc. Natl. Acad. Sci. USA.

[13] Makino S, Sasakawa C, Kamata K, Kurata T, Yoshikawa M (1986). A genetic determinant required for continuous reinfection of adjacent cells on large plasmid in *S. flexneri* 2a. Cell.

[14] Suzuki T (2002). Neural Wiskott-Aldrich syndrome protein (N-WASP) is the specific ligand for *Shigella* VirG among the WASP family and determines the host cell type allowing actin-based spreading. Cell Microbiol..

[15] Egile C (1999). Activation of the CDC42 effector N-WASP by the *Shigella flexneri* IcsA protein promotes actin nucleation by Arp2/3 complex and bacterial actin-based motility. J. Cell Biol..

[16] Dragoi AM, Agaisse H (2014). The serine/threonine kinase STK11 promotes *Shigella flexneri* dissemination through establishment of cell–cell contacts competent for tyrosine kinase signaling. Infect. Immun..

[17] Dragoi AM, Agaisse H (2015). The class II phosphatidylinositol 3-phosphate kinase PIK3C2A promotes *Shigella flexneri* dissemination through formation of vacuole-like protrusions. Infect. Immun..

[18] Gouin E (1999). A comparative study of the actin-based motilities of the pathogenic bacteria *Listeria monocytogenes*, *Shigella flexneri* and *Rickettsia conorii*. J. Cell Sci..

[19] Kadurugamuwa JL, Rohde M, Wehland J, Timmis KN (1991). Intercellular spread of *Shigella flexneri* through a monolayer mediated by membranous protrusions and associated with reorganization of the cytoskeletal protein vinculin. Infect. Immun..

[20] Agaisse H (2016). Molecular and cellular mechanisms of *Shigella flexneri* dissemination. Front Cell Infect. Microbiol..

[21] Freter R (1956). Experimental enteric *Shigella* and *Vibrio* infections in mice and guinea pigs. J. Exp. Med..

[22] Rabbani GH (1995). Development of an improved animal model of shigellosis in the adult rabbit by colonic infection with *Shigella flexneri* 2a. Infect. Immun..

[23] Sereny B (1957). Experimental keratoconjunctivitis shigellosa. Acta Microbiol. Acad. Sci. Hung..

[24] Shim DH (2007). New animal model of shigellosis in the Guinea pig: its usefulness for protective efficacy studies. J. Immunol..

[25] Voino-Yasenetsky MV, Voino-Yasenetskaya MK (1962). Experimental pneumonia caused by bacteria of the *Shigella* group. Acta Morphol. Acad. Sci. Hung..

[26] Yang JY (2014). A mouse model of shigellosis by intraperitoneal infection. J. Infect. Dis..

[27] Ritchie, J. M., Rui, H., Bronson, R. T. & Waldor, M. K. Back to the future: studying cholera pathogenesis using infant rabbits. *MBio* **1**, 10.1128/mBio.00047-10 (2010). 10.1128/mBio.00047-10 PMC2912669 20689747

[28] Ritchie JM, Waldor MK (2005). The locus of enterocyte effacement-encoded effector proteins all promote enterohemorrhagic *Escherichia coli* pathogenicity in infant rabbits. Infect. Immun..

[29] Kuehl CJ, Dragoi AM, Agaisse H (2014). The *Shigella flexneri* type 3 secretion system is required for tyrosine kinase-dependent protrusion resolution, and vacuole escape during bacterial dissemination. PLoS ONE.

[30] Perdomo OJ (1994). Acute inflammation causes epithelial invasion and mucosal destruction in experimental shigellosis. J. Exp. Med..

[31] Sansonetti PJ, Arondel J, Cavaillon JM, Huerre M (1995). Role of interleukin-1 in the pathogenesis of experimental shigellosis. J. Clin. Invest..

[32] Sansonetti PJ, Arondel J, Huerre M, Harada A, Matsushima K (1999). Interleukin-8 controls bacterial transepithelial translocation at the cost of epithelial destruction in experimental shigellosis. Infect. Immun..

[33] Sansonetti PJ, Arondel J, Fontaine A, d’Hauteville H, Bernardini ML (1991). *OmpB* (osmo-regulation) and *icsA* (cell-to-cell spread) mutants of *Shigella flexneri*: vaccine candidates and probes to study the pathogenesis of shigellosis. Vaccine.

[34] Wassef JS, Keren DF, Mailloux JL (1989). Role of M cells in initial antigen uptake and in ulcer formation in the rabbit intestinal loop model of shigellosis. Infect. Immun..

[35] Zhang Z, Jin L, Champion G, Seydel KB, Stanley SL (2001). *Shigella* infection in a SCID mouse-human intestinal xenograft model: role for neutrophils in containing bacterial dissemination in human intestine. Infect. Immun..

