# Supplementary material for: Critical role of bacterial dissemination in an infant rabbit model of bacillary dysentery
Source: Nat Commun. 2019 Apr 23;10:1826. doi: 10.1038/s41467-019-09808-4 (PMC6478941; doi:10.1038/s41467-019-09808-4)
Supplement: Supplementary file 1 — Supplementary Information [file 41467_2019_9808_MOESM1_ESM.pdf]

Supplementary Information

Critical role of bacterial dissemination in an infant rabbit model of bacillary dysentery

Yum et al.

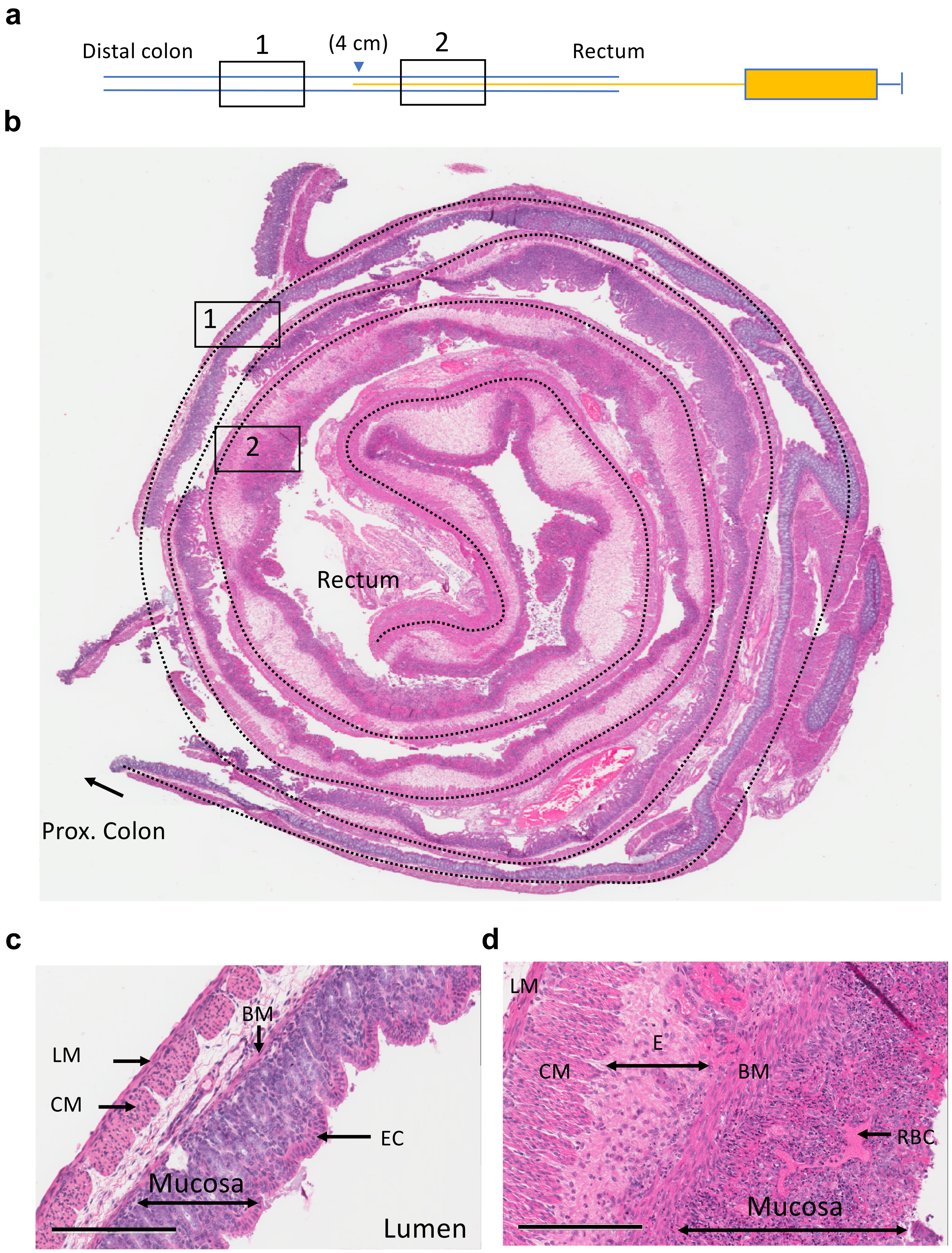

Supplementary Figure 1

Supplementary Figure 1. Experimental procedures in the infant rabbit model of bacillary dysentery. (**a**) The inoculum is delivered in the distal colon by rectal inoculation with a feeding tube, 4 cm away from the rectum. (**b**) Representative image showing a swiss-roll of the distal colon with healthy non-infected tissue (box 1) and infected tissue (box 2). Box 1 and box 2, as shown in (a). (**c,d**) High-magnification images corresponding to the area shown in box 1 (c) and box 2 (d). Scale bars, 200 µm. LM, longitudinal muscles; CM, circular muscles; BM, basement membrane, EC, epithelial cells; E, edema; RBC, red blood cells. In (d), note (i) the sub-mucosal (connective tissue) and CM edema, (ii) the vascular lesions as reflected by the presence of RBC in the mucosa and (iii) the epithelial fenestration as reflected by the absence of EC in the infected area of the distal colon (box 2).

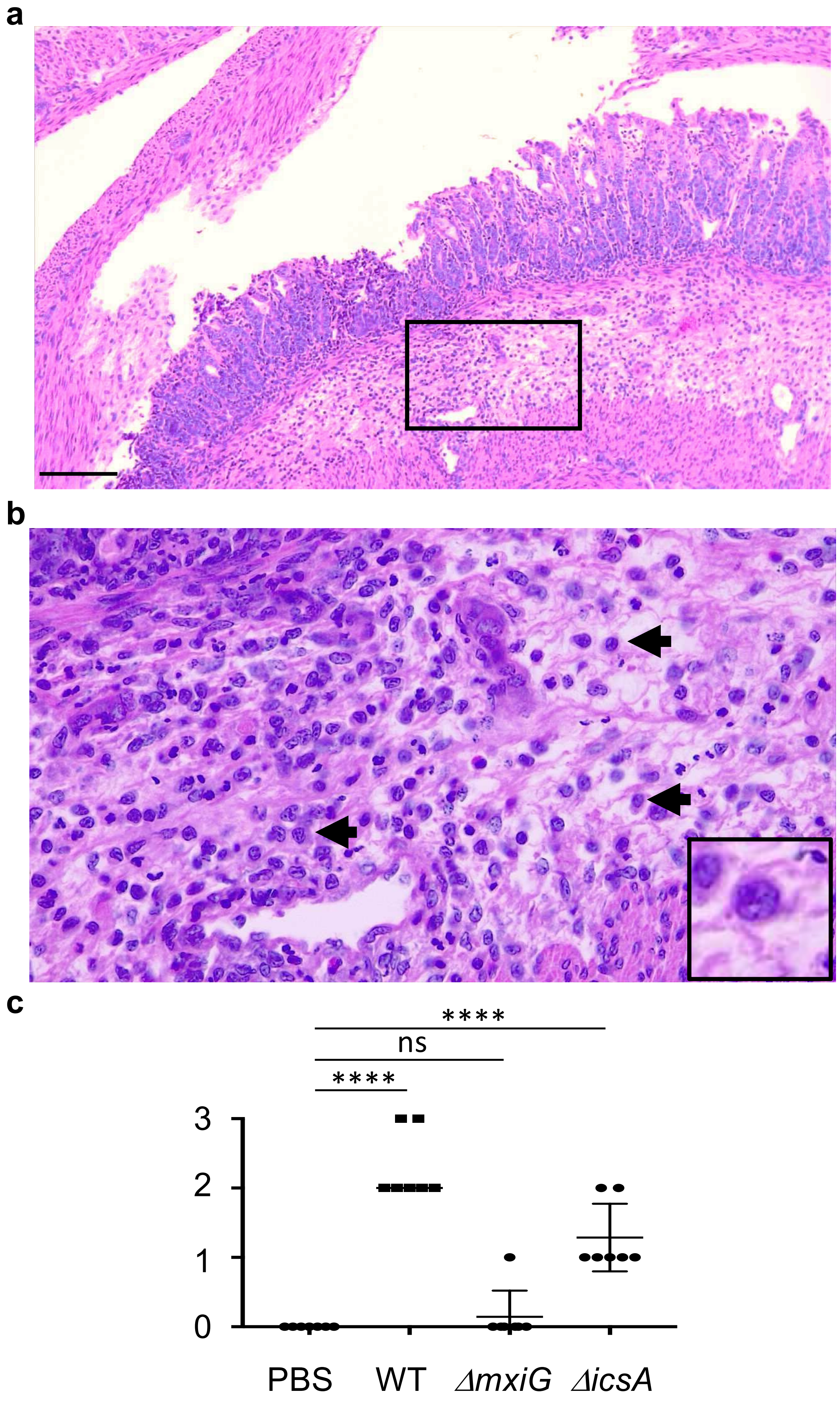

Supplementary Figure 2

Supplementary Figure 2. Monocyte infiltration in the colon of infant rabbit infected with *S. flexneri*. (**a**) Representative images of the infected area (10X). Scale bar, 100 µm. (**b**) High-magnification image (40X) of the boxed area in (a) showing examples of monocytes (arrows). Inset, zoom-in on the monocyte indicated by the right arrow in (b). (**c**) Histopathology scores of monocyte infiltration in the distal colon of mock-treated (PBS) animals, and animals infected with the *ΔmxiG* or the *ΔicsA* mutants, or the wild type strain (WT). Statistical analysis, one-way ANOVA. PBS vs. WT, $P<0.0001$; PBS vs. *ΔmxiG*, ns; PBS vs. *ΔicsA*, $P<0.0001$.

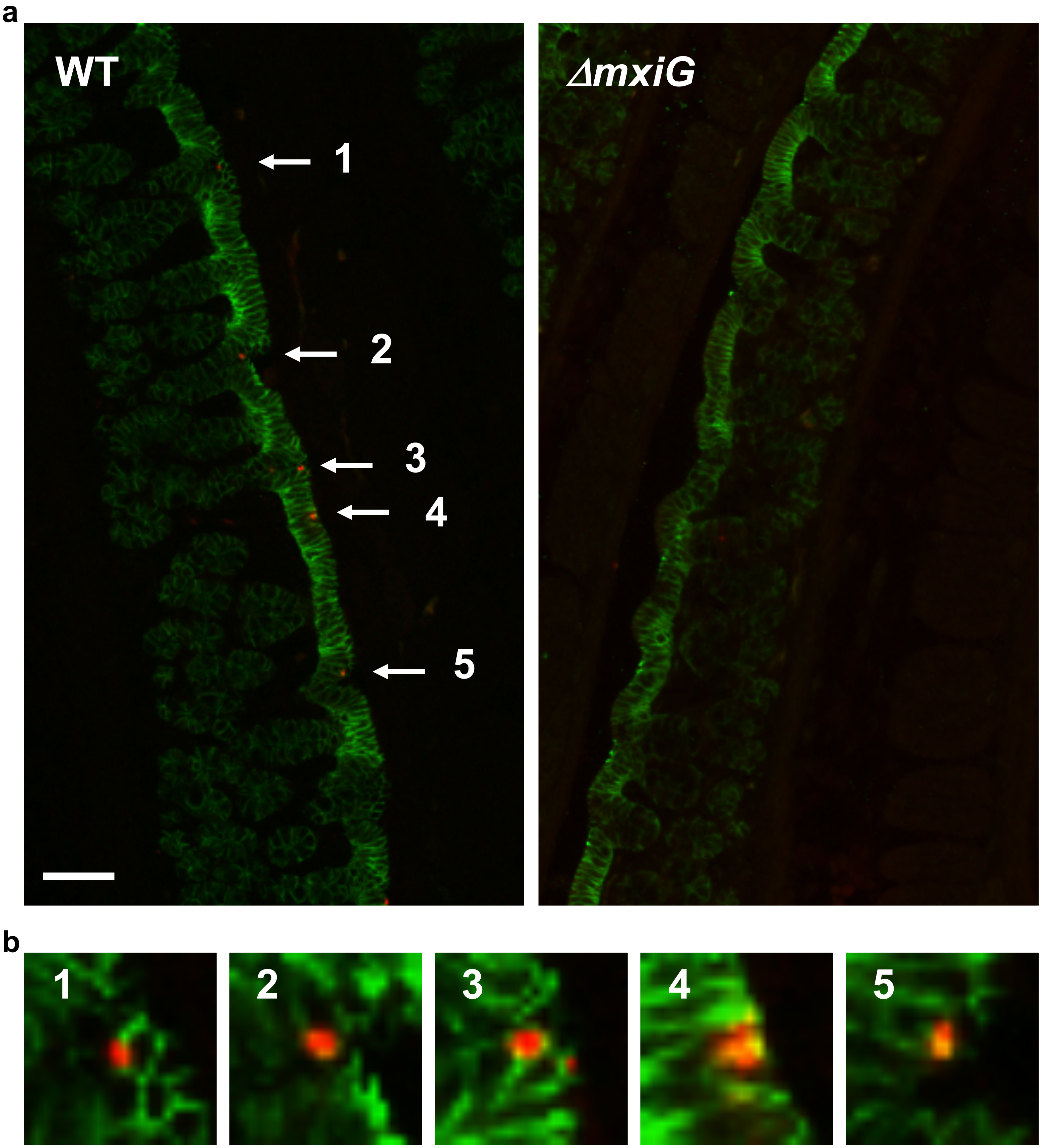

Supplementary Figure 3

Supplementary Figure 3. The *ΔmxiG* mutant is not invasive. (**a**) Representative images of immunofluorescence stains for E-cadherin (green) and *S. flexneri* (red) in the distal colon of animals infected with wild type *S. flexneri* (left) or the *ΔmxiG* mutant (right). Arrows indicate intracellular bacteria. Scale bar, 100 µm. (**b**) Zoom-in images corresponding to the area indicated by the arrows in (a). Note the absence of bacteria associated with the E-cadherin positive cells in the sample infected with the *ΔmxiG* mutant.

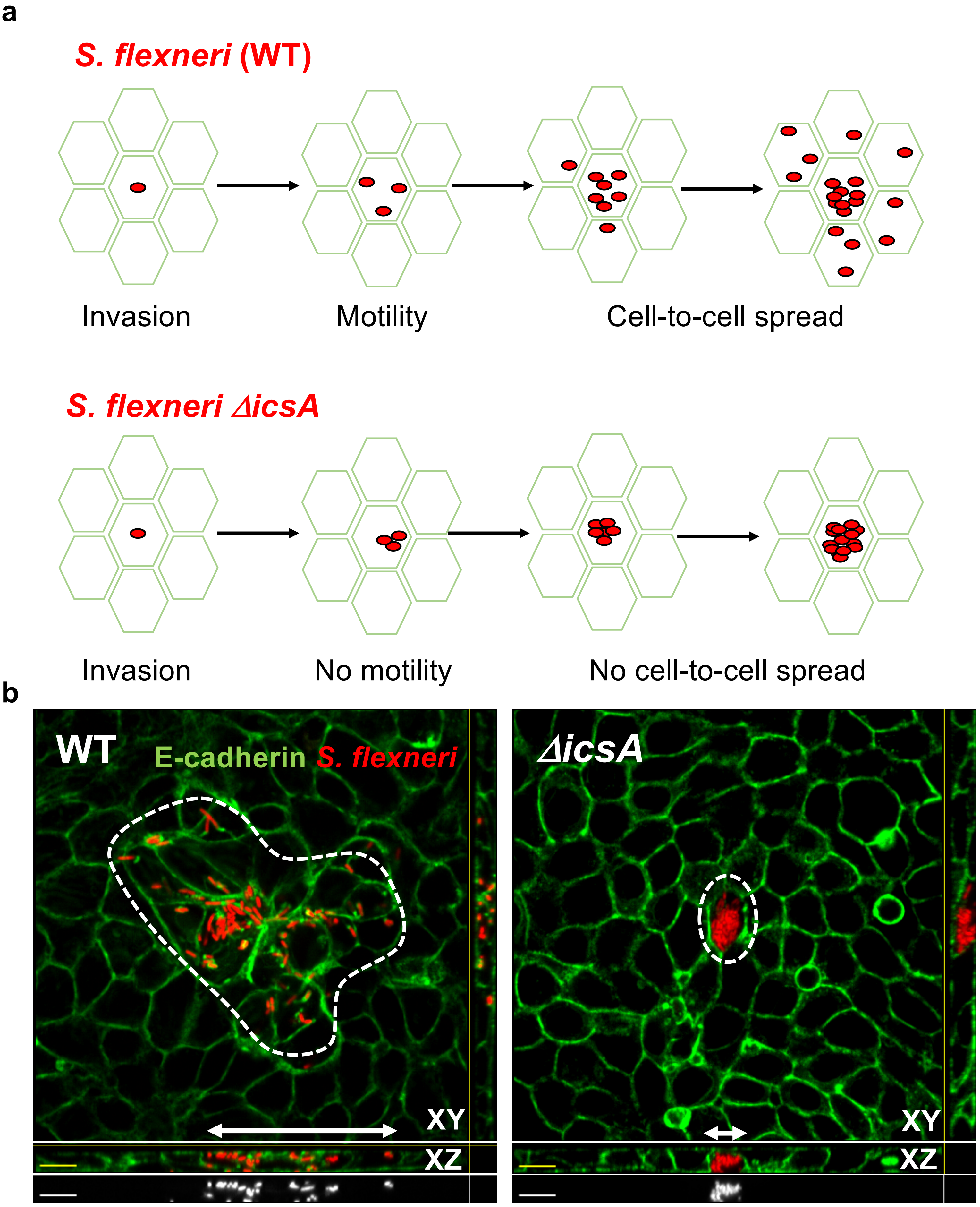

Supplementary Figure 4

Supplementary Figure 4. Features of cell-to-cell spread in tissue culture cells. (**a**) Schematic depicting the outcome of infection with wild type (WT) *S. flexneri* (top) or the *ΔicsA* mutant (bottom). The *ΔicsA* mutant is as invasive as wild type *S. flexneri* (Red, Invasion). While wild type bacteria display actin-based motility (Red, motility), the actin-based motility defective *ΔicsA* mutant (Red, No motility) grows as a micro-colony. Wild type bacteria spread to adjacent cells and form infection foci (Red, Cell-to-cell spread). The *ΔicsA* mutant grows in primarily infected cells, but does not spread to adjacent cells (Red, No cell-to-cell spread). (**b**) Representative images of the outcome of infection with wild type (WT) *S. flexneri* (left) or the *ΔicsA* mutant (right) in HT-29 cells. E-cadherin, green; bacteria, red. The dotted lines delineate the infected area showing formation of an infection focus and single cell infection with wild type *S. flexneri* and the *ΔicsA* mutant, respectively. The double arrows indicate the spread of the bacteria in the XZ dimension. Scale bars, 10 μm.

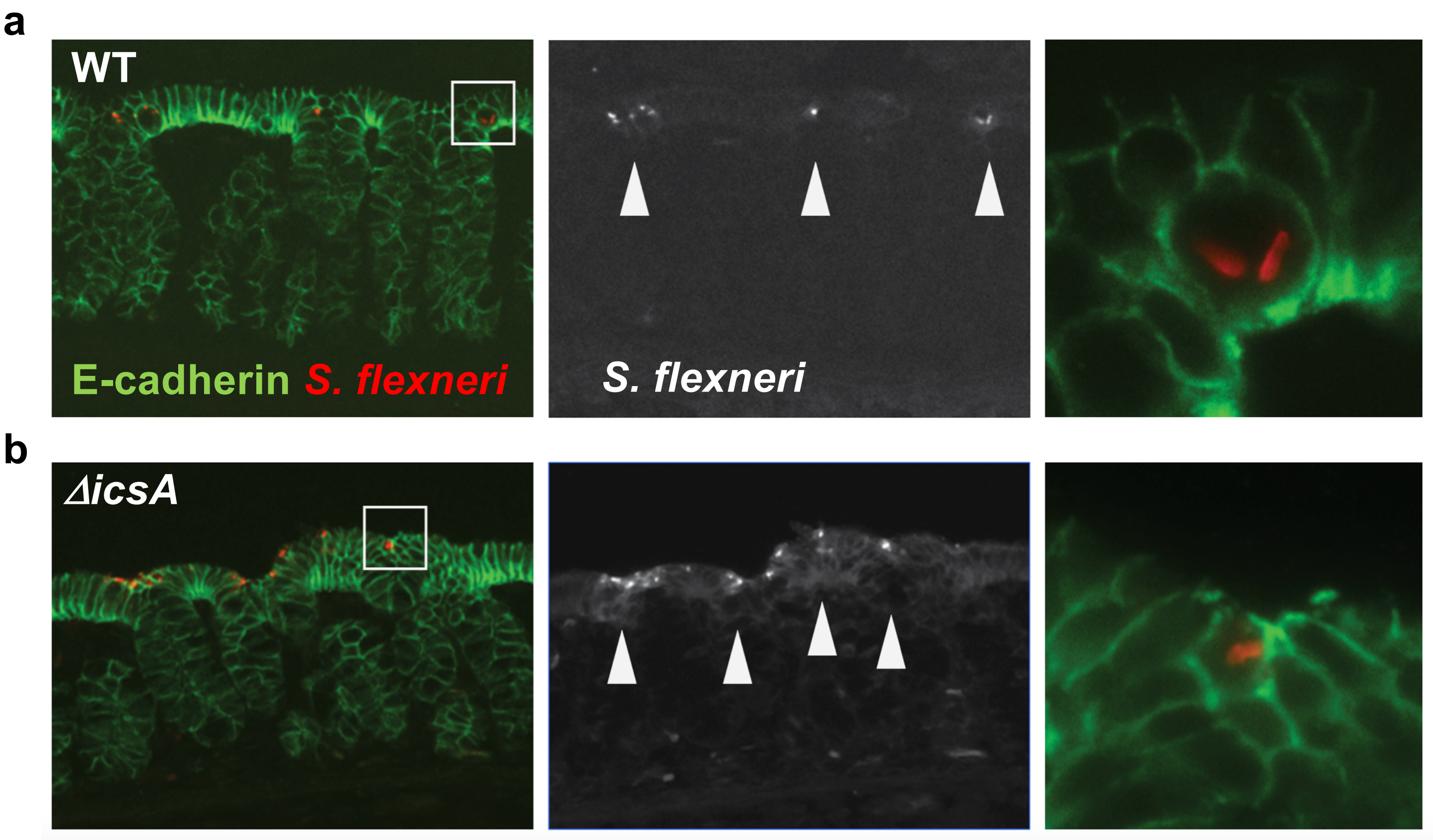

Supplementary Figure 5. The *ΔicsA* mutant is as invasive as wild type. (**a,b**) Representative images of the distal colon of animals infected with wild type (WT) (a) or the *ΔicsA* mutant (b) 2 hours pi. E-cadherin, green; bacteria, red. Left panels, merge; middle panels, bacteria only. Right panels, high-magnification images of the boxed area in the left panel showing intracellular bacteria in E-cadherin positive epithelial cells. Arrowheads in the middle panels indicate intracellular bacteria.

Supplementary Figure 5

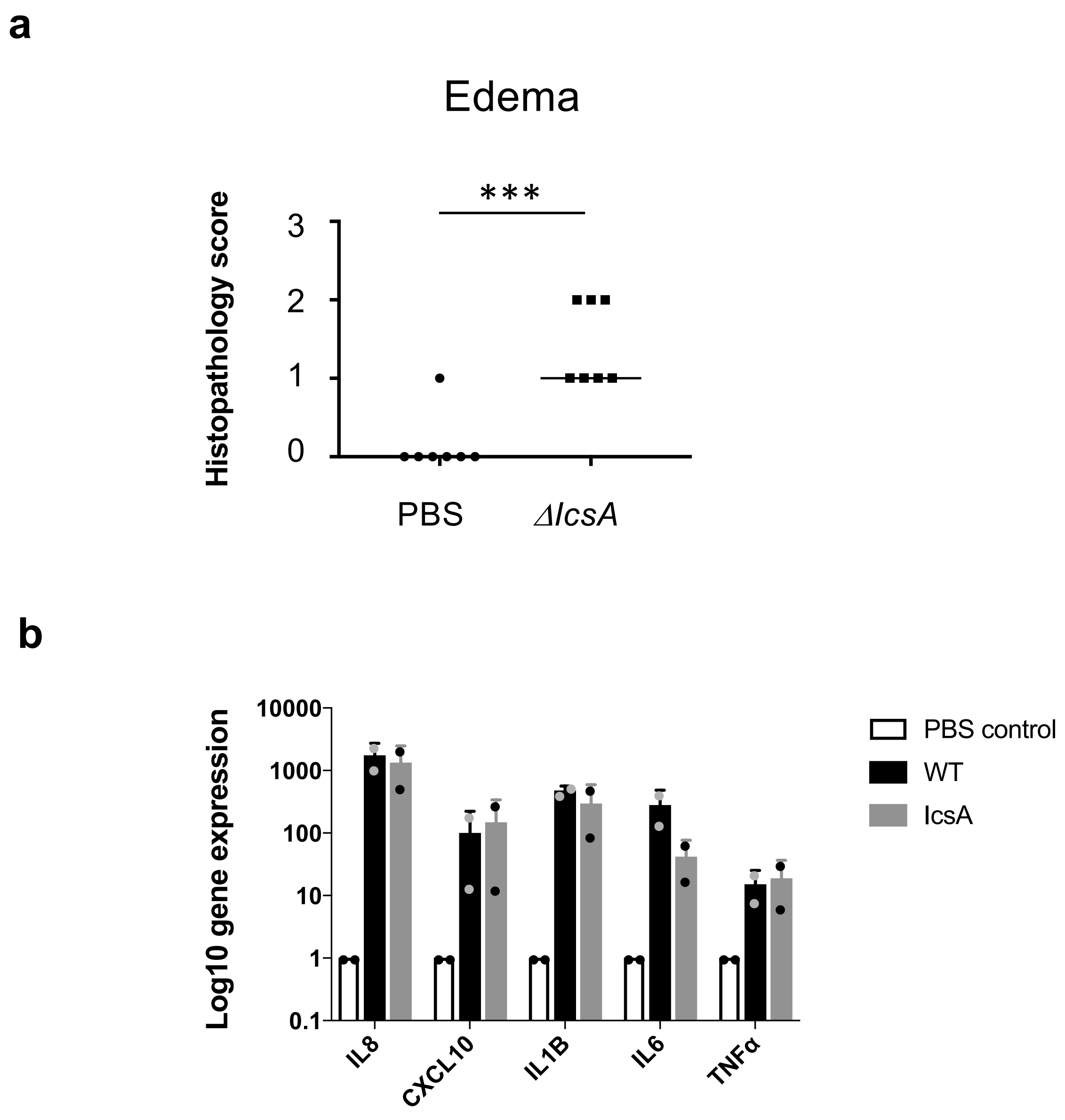

Supplementary Figure 6. Pro-inflammatory immune responses in the colon of animals infected with the *ΔicsA* mutant. (**a**) Histopathology scores of edema (E). Statistical analysis, unpaired t-test. *ΔicsA* vs. PBS, P<0.0005. (**b**) Graph showing chemokine (IL-8 and CXCL10) and cytokine (IL1B, IL6 and TNFα) gene expression with respect to levels observed in mock-treated animals (PBS) in the distal colon of animals 8 hours pi with wild type *S. flexneri* or the *ΔicsA* mutant. Overlay dot plots represent individual data points. Error bars represent standard deviation of the mean.

Supplementary Figure 6

Score	Vascular lesions
0	No change
1	Mild focal to multifocal vascular congestion
2	Moderate focal to multifocal vascular congestion and mild to moderate focal to multifocal mucosal hemorrhage
3	Marked multifocal to diffuse vascular congestion and moderate to marked multifocal to diffuse mucosal hemorrhage

Score	Sub-mucosal edema
0	No change
1	Mild (10-20%)
2	Moderate (20-40%)
3	Marked/Severe (>40%)

	Epithelial fenestration
	% Fenestration = (length of colonic epithelium with signs of fenestration/total length of colonic epithelium)*100

Score	Heterophil infiltration
0	No change
1	No change (0-5 heterophils/ high power field)
2	Moderate multifocal infiltrate (21-40 heterophils/ high power field)
3	Marked multifocal infiltrate (> 40 heterophils/ high power field)

Score	Monocyte infiltration
0	No change
1	No change (0-5 monocytes/ high power field)
2	Moderate multifocal infiltrate (21-40 monocytes / high power field)
3	Marked multifocal infiltrate (> 40 monocytes / high power field)

Supplementary Table 1. Histopathology scoring system.

Gene name	Sequence	Universal Library Probe Number
GAPDH left	GATCCCGCCAACATCAAGT	104
GAPDH right	CTCCATGGTGGTGAAGACG	
IL-8 left	TTAAATACTGCATGGATCTGTCG	27
IL-8 right	TTCGGGTTTAACTGAGAATCCTA	
CXCL10 left	CCTGCAAGTCAATCTTGTGC	7
CXCL10 right	CTTGATGGCTTTCAATTCTGG	
IL-1β left	CTGCGGCAGAAAGCAGTT	97
IL-1β right	GAAAGTTCTCAGGCCGTCAT	
IL-6 left	GTCCTGCTTGCGGAATTT	13
IL-6 right	AATGGACAGGATGGTGTGTTC	

Supplementary Table 2. Primers used for real-time PCR studies of immune gene expression.
