# Supplementary material for: Critical role of bacterial dissemination in an infant rabbit model of bacillary dysentery
Source: Nat Commun. 2019 Apr 23;10:1826. doi: 10.1038/s41467-019-09808-4 (PMC6478941; doi:10.1038/s41467-019-09808-4)
Supplement: Supplementary file 2 — Reporting Summary [file 41467_2019_9808_MOESM2_ESM.pdf]

nature research

Corresponding author(s): Hervé Agaisse

Last updated by author(s): Feb 24, 2019

Reporting Summary

Nature Research wishes to improve the reproducibility of the work that we publish. This form provides structure for consistency and transparency in reporting. For further information on Nature Research policies, see Authors & Referees and the Editorial Policy Checklist.

Statistics

For all statistical analyses, confirm that the following items are present in the figure legend, table legend, main text, or Methods section.

n/a	Confirmed	
☐	☒	The exact sample size (*n*) for each experimental group/condition, given as a discrete number and unit of measurement
☐	☒	A statement on whether measurements were taken from distinct samples or whether the same sample was measured repeatedly
☐	☒	The statistical test(s) used AND whether they are one- or two-sided<br>*Only common tests should be described solely by name; describe more complex techniques in the Methods section.*
☒	☐	A description of all covariates tested
☒	☐	A description of any assumptions or corrections, such as tests of normality and adjustment for multiple comparisons
☐	☒	A full description of the statistical parameters including central tendency (e.g. means) or other basic estimates (e.g. regression coefficient) AND variation (e.g. standard deviation) or associated estimates of uncertainty (e.g. confidence intervals)
☒	☐	For null hypothesis testing, the test statistic (e.g. *F*, *t*, *r*) with confidence intervals, effect sizes, degrees of freedom and *P* value noted<br>*Give P values as exact values whenever suitable.*
☒	☐	For Bayesian analysis, information on the choice of priors and Markov chain Monte Carlo settings
☒	☐	For hierarchical and complex designs, identification of the appropriate level for tests and full reporting of outcomes
☒	☐	Estimates of effect sizes (e.g. Cohen's *d*, Pearson's *r*), indicating how they were calculated

Our web collection on statistics for biologists contains articles on many of the points above.

Software and code

Policy information about availability of computer code

Data collection	Image collection was performed using the MetaMorph software (Molecular Devices) and the LAS X software (Leica).
Data analysis	Statistical analysis of the data was conducted with the PRISM software (GraphPad).

For manuscripts utilizing custom algorithms or software that are central to the research but not yet described in published literature, software must be made available to editors/reviewers. We strongly encourage code deposition in a community repository (e.g. GitHub). See the Nature Research guidelines for submitting code & software for further information.

Data

Policy information about availability of data

All manuscripts must include a data availability statement. This statement should provide the following information, where applicable:

- Accession codes, unique identifiers, or web links for publicly available datasets
- A list of figures that have associated raw data
- A description of any restrictions on data availability

All data generated or analyzed during this study are included in this published article (and its supplementary information files).

Field-specific reporting

Please select the one below that is the best fit for your research. If you are not sure, read the appropriate sections before making your selection.

☒ Life sciences ☐ Behavioural & social sciences ☐ Ecological, evolutionary & environmental sciences

For a reference copy of the document with all sections, see nature.com/documents/nr-reporting-summary-flat.pdf

Life sciences study design

All studies must disclose on these points even when the disclosure is negative.

Sample size	The minimum sample size for histopathology analysis is 5 animals per condition. We used a sample size of 7.
Data exclusions	No data were excluded from the analyses.
Replication	The histopathology experiments were conducted in three sets of independent experiments and the results were pooled for statistical analyses.
Randomization	Allocation of infection groups was random.
Blinding	The scoring of the histopathogy sections was blinded.

Reporting for specific materials, systems and methods

We require information from authors about some types of materials, experimental systems and methods used in many studies. Here, indicate whether each material, system or method listed is relevant to your study. If you are not sure if a list item applies to your research, read the appropriate section before selecting a response.

Materials & experimental systems

n/a	Involved in the study
☐	☒ Antibodies
☒	☐ Eukaryotic cell lines
☒	☐ Palaeontology
☐	☒ Animals and other organisms
☒	☐ Human research participants
☒	☐ Clinical data

Methods

n/a	Involved in the study
☒	☐ ChIP-seq
☒	☐ Flow cytometry
☒	☐ MRI-based neuroimaging

Antibodies

Antibodies used	Primary antibodies: mouse anti E-cadherin , BD Biosciences (#610181); rabbit anti Shigella sp., ViroStat (#0901). Secondary antibodies: goat anti-Mouse Alexa Fluor 514, Thermo Fisher Scientific (#A-31555); goat anti-Rabbit Pacific Blue: Thermo Fisher Scientific (#P10994)
Validation	The specificity of the E-cadherin antibody was validated by our lab through gene silencing and western blotting. The specificity of the Shigella antibody was demonstrated by the absence of signal in non-infected colon.

Animals and other organisms

Policy information about studies involving animals; ARRIVE guidelines recommended for reporting animal research

Laboratory animals	Infant rabbits, New Zealand white, 10-15-day old, born from pregnant females purchased from Charles-River.
Wild animals	This study did not involve wild animals.
Field-collected samples	This study did not involve samples collected from the field.
Ethics oversight	The animal protocol was approved by the IACUC at the University of Virginia.

Note that full information on the approval of the study protocol must also be provided in the manuscript.
